# Modelling severe *Staphylococcus aureus* sepsis in conscious pigs: are implications for animal welfare justified?

**DOI:** 10.1186/s13104-016-1888-7

**Published:** 2016-02-16

**Authors:** Helle G. Olsen, Mads Kjelgaard-Hansen, Pernille Tveden-Nyborg, Malene M. Birck, Karsten P. Hammelev, Andreas Vegge, Bent Aalbæk, Páll S. Leifsson, Henrik E. Jensen, Tine Iburg, Peter M. H. Heegaard, Ole L. Nielsen

**Affiliations:** Department of Veterinary Disease Biology, Faculty of Health and Medical Sciences, University of Copenhagen, Frederiksberg, Denmark; Department of Veterinary Clinical and Animal Sciences, Faculty of Health and Medical Sciences, University of Copenhagen, Frederiksberg, Denmark; Department of Experimental Medicine, Faculty of Health and Medical Sciences, University of Copenhagen, Frederiksberg, Denmark; Department of Nutrition, Exercise and Sports, Faculty of Science, University of Copenhagen, Frederiksberg, Denmark; National Veterinary Institute, Technical University of Denmark, Frederiksberg, Denmark; Novo Nordisk, Måløv, Denmark

**Keywords:** Sepsis, Pigs, Animal model, Galactose elimination capacity (GEC), Liver dysfunction, Humane endpoints, Animal welfare

## Abstract

**Background:**

A porcine model of haematogenous *Staphylococcus aureus* sepsis has previously been established in our research group. In these studies, pigs developed severe sepsis including liver dysfunction during a 48 h study period. As pigs were awake during the study, animal welfare was challenged by the severity of induced disease, which in some cases necessitated humane euthanasia. A pilot study was therefore performed in order to establish the sufficient inoculum concentration and application protocol needed to produce signs of liver dysfunction within limits of our pre-defined humane endpoints.

**Methods:**

Four pigs received 1 × 10^8^ cfu/kg BW of *S. aureus*, and two controls were sham inoculated with saline. A fixed infusion rate of 3 mL/min was used, while the inoculum concentration, i.e., the dose volume, was changed between the pigs. The following dose volumes were used: 10 mL (n = 1), 20 mL (n = 2), and 30 mL (n = 1), corresponding to infusion durations of 3.33, 6.66, and 10 min at dose rates of 3 × 10^7^, 1.5 × 10^7^, and 1 × 10^7^ cfu/min/kg BW, respectively. Blood samples were drawn for complete blood count, clinical chemistry, and inflammatory markers before and every 6 h after inoculation. Prior to euthanasia, a galactose elimination capacity test was performed to assess liver function. Pigs were euthanised 48 h post inoculation for necropsy and histopathological evaluation.

**Results:**

While infusion times of 6.66 min, and higher, did not induce liver dysfunction (n = 3), the infusion time of 3.33 min (n = 1) caused alterations in parameters similar to what had been seen in our previous studies, i.e., increasing bilirubin and aspartate aminotransferase, as well as histopathological occurrence of intravascular fibrin split products in the liver. This pig was however euthanised after 30 h, according to humane endpoints.

**Conclusions:**

A usable balance between scientific purpose and animal welfare could not be achieved, and we therefore find it hard to justify further use of this conscious porcine sepsis model. In order to make a model of translational relevance for human sepsis, we suggest that future model versions should use long-term anaesthesia.

## Background

The liver plays a central role in sepsis, being a multifunctional organ. Within human intensive care units (ICUs), cholestatic liver dysfunction is a frequent incidence which may arise from multiple factors such as infection, sepsis, major surgery, blood transfusions, parenteral nutrition, and use of antibiotics and other drugs with hepatotoxic side effects [1–3]. The diagnosis is commonly based on increased serum concentrations of bilirubin, supported by increases in liver enzymes such as the transaminases aspartate aminotransferase (AST) and alanine aminotransferase (ALT), alkaline phosphatase (AP), lactate dehydrogenase, and/or occurrence of hypoalbuminemia [4]. Persistent hyperbilirubinemia is associated with increased mortality [5, 6], also in patients with *Staphylococcus aureus* sepsis [7]. The pathogenesis of sepsis-related liver dysfunction is however not well understood [2, 3].

The aims of the study reported in this paper were twofold: (1) We have previously performed two related sepsis model experiments in pigs inoculated hematogenously with *S. aureus* [8, 9], with a study duration of up to 48 h. The pigs were at a conscious state (i.e., un-anaesthetised) during the observation period from inoculation to euthanasia. Clinical signs of sepsis included increased body temperature, neutrophilia, increased C-reactive protein (CRP) and interleukin-6 (IL-6), and decreased serum iron. Increasing hypercoagulability and decreasing numbers of platelets indicated dysfunction of the blood clotting system, and of special note, liver dysfunction was indirectly indicated by hyperbilirubinemia, increased AST together with normal levels of creatine kinase (CK), as well as fibrin exudation from small vessels within the liver. There were few to none bacteria cultured from liver tissue, suggesting that the hepatic affection was part of a systemic inflammatory response rather than due to local hepatic infection [8, 9]. These observations led to the hypothesis that pigs with experimental *S. aureus* sepsis develop liver dysfunction. The first objective of this study was therefore to establish a method with which we could confirm and quantify presence of liver dysfunction in pigs 48 h after hematogeneous inoculation with *S. aureus*, i.e., by introducing the galactose elimination capacity test (GEC) to the experimental setup. (2) Animal welfare and model robustness however remained a challenge in the study design: some pigs responded with apnoea and cardiac arrest during the inoculation procedure, and other pigs were euthanised before scheduled due to development of severe clinical disease, i.e., respiratory impairment from acute, severe pneumonia [8, 9]. The inoculum concentration has previously been suggested to play a role in directing the severity of sepsis in this model [10]. We used this observation to form our second hypothesis for this study, namely that the rate [cfu/min/kg body weight (BW)] of infusion of *S. aureus* is determining for the degree of induced clinical disease. In order to accomplish the first objective regarding quantification of liver dysfunction, the second objective of the study was therefore to establish the inoculum concentration and application protocol needed to produce signs of liver dysfunction without causing adverse effects during inoculation and without reaching pre-defined humane endpoints during the 48 h study period.

In brief, this article reports that re-construction of signs of liver dysfunction could not be achieved within the limits of our pre-defined humane endpoints, and a usable balance between scientific objectives and animal welfare therefore seems difficult to establish for this conscious porcine sepsis model.

## Methods

### Animals

The study was carried out in accordance with a protocol approved by the Danish Animal Experiments Inspectorate (license no. 2012-15-2934-00702), in compliance with Directive 2010/63/EU of the European Parliament and of the council of 22 September 2010 on the protection of animals used for scientific purposes [11].

Six female, juvenile, crossbred pigs (Danish Landrace/Yorkshire/Duroc) from a specific pathogen free pig herd were used. The sample size was determined based on convenience sampling. At arrival, *S. aureus* was recovered from the nasal cavity or perineum from three out of six pigs (Case-3, Case-4, and Control-2), indicating environmental exposure prior to commencement of this experiment (method provided under the heading “Pathology and bacteriology“).

The pigs were regarded as clinically healthy based on evaluation of standard haematology and biochemistry results from blood samples obtained at arrival, as well as on daily visual inspections during the acclimatisation period of 8–19 days. The pigs were fed a commercial swine feed and housed in groups during the acclimatisation period. Body weight (BW) was 30‒36 kg at the day of inoculation.

### Establishment of permanent IV access

One to 3 days before inoculation, pigs were premedicated intramuscularly with a combination of the following drugs: 0.83 mg/kg BW zolazepam and 0.83 mg/kg BW tiletamine (Zoletil 50/50^®^ vet; Virbac, Carros, France), 0.83 mg/kg BW xylazine (Narcoxyl^®^ 20 mg/mL; Intervet, Boxmeer, Netherlands), 0.83 mg/kg BW ketamine (Ketaminol^®^ 100 mg/mL; Intervet, Boxmeer, Netherlands), and 0.17 mg/kg BW butorphanol (Torbugesic^®^ 10 mg/mL; ScanVet, Fredensborg, Denmark). This premedication combination was recommended by the local laboratory animal facility staff who use it routinely for pigs. The pigs were shifted to IV propofol infusion (6 mg/kg BW/h; Rapinovet^®^ vet 10 mg/mL; Schering-Plough Animal Health, Ballerup, Denmark) as soon as IV access was gained (see below).

A permanent central vein catheter (BD Careflow 3 or 4 Fr, 20 cm, Cat.nos. 681643 or 681649; BD, NJ, USA) was aseptically inserted through the auricular vein of each ear, and guided into the jugular vein. The catheter was fixed to the ear pinna with sutures (poly-filament, non-absorbable Dagrofil suture 0; B.Braun, Tuttlingen, Germany) and adhesive bandages. To prevent clotting of the catheters, 2.5–3.5 mL saline with heparin (1000 IE/mL) (Heparin Leo^®^ 5000 IE; Leo Pharma, Ballerup, Denmark) was deposited as lock solution. The pigs were allowed to recover from anaesthesia and subsequently housed individually in biosecurity level 2 stables for the remainder of the study period.

### Inoculation and experimental design

During the inoculation procedure 1–3 days after establishment of IV access, pigs were anaesthetised for the second time using the same premedication and anaesthetic protocol as described for the IV access procedure above. The pigs were intubated and spontaneously breathing atmospheric air supplied with oxygen from a hose placed near the tube opening (100 % oxygen, 0.5–1.0 L/min) and monitored with echocardiography and pulse oximetry.

**Fig. 1:**
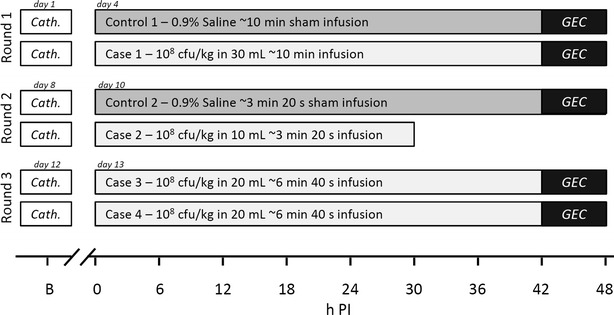
Study overview. The study was conducted in three consecutive rounds over 15 days. *B* indicates baseline blood samples which were obtained at the IV catheterisation procedure (cath.), i.e., 1–3 days before inoculation. *GEC* galactose elimination capacity test

The experiment was carried out in three consecutive rounds, with one inoculated pig and one control pig in each of the first two rounds and two inoculated pigs in the third round (Fig. 1). The allocation was based on convenience sampling.

A swine-specific strain of *S. aureus* (isolate S54F9) [12, 13] was infused through the left auricular IV catheter in four pigs at a dose of 1 × 10^8^ cfu/kg BW suspended in 0.9 % NaCl. The infusion time corresponded to dose volumes of 30, 10, and 20 mL in rounds one, two and three, respectively, using a fixed infusion rate of 3 mL/min (Alaris CC Plus Syringe Pump). Infusion durations were thus 10, 3.33, and 6.66 min, respectively, which corresponded to inoculum dose rates of 1 × 10^7^, 3 × 10^7^, and 1.5 × 10^7^ cfu/min/kg BW. Inoculum concentrations were 3.33 × 10^6^, 1 × 10^7^, and 5 × 10^6^ cfu/mL/kg BW. Two control pigs were infused with volumes of saline (0.9 % NaCl) corresponding to their parallel inoculated cases (Fig. 1). *S. aureus* inoculum was prepared as previously described [14].

### Observation period following inoculation

After completion of inoculation, pigs were allowed to recover from anaesthesia, while a self-retracting swivel line was attached to a harness on the pig (Trixie dog harness, size L, Tarp, Germany) [15], allowing the pig to move around freely. Via this line, a connector tube was led from the right ear vein catheter and outside the pen, enabling permanent vascular access for repeated blood sampling and infusion of IV analgesics without requiring to restrain the pig. A constant infusion of isotonic saline (0.9 % NaCl; 3 mL/h) was set to keep the vein open, and only interrupted during blood sampling or infusion of analgesics.

The pigs were observed frequently during the experimental study period (0–6 h intervals). Clinical examinations (e.g. body temperature measurements, auscultations, oxygen saturation measurements) were only made on indication, i.e., when a pig’s clinical condition was judged as deteriorating or distressing. Buprenorphin, 0.01‒0.02 mg/kg BW (Temgesic^®^ 0.3 mg/mL, Reckitt Benckiser, United Kingdom) was administered IV to all inoculated pigs and control pigs every 6 h throughout the experimental study period.

### Humane endpoints

Humane endpoints were based on the ones used in previous sepsis studies conducted by our group [8, 9], and were elaborated further to reduce subjectivity in the assessment of animal welfare. In short, the endpoints included: (1) Signs of severe dyspnoea, defined as an inability of the pig to keep oxygen saturation above 90 %. (2) Inability to get up and/or move. (3) Signs indicating encephalitis, e.g. nystagmus, paresis, or head tilt.

### Blood measurements

Baseline blood samples (B) were drawn from the IV catheter 1–3 days before inoculation (Fig. 1). Starting just prior to inoculation (0 h PI), blood was further sampled every 6 h throughout the remaining study period.

Haematology was performed using EDTA stabilized whole blood (ADVIA 120 analyzer, Bayer Healthcare Diagnostics). Clinical chemistry analyses were performed on serum (ADVIA 1650, Bayer Healthcare Diagnostics). C-reactive protein was analysed using a dendrimer-coupled cytidine diphosphocholine sandwich enzyme-linked immunosorbent assay (ELISA) [16]. Serum amyloid A (SAA) was measured with a sandwich ELISA (Phase SAA assay, Tridelta Development Ltd.) as originally described by McDonald et al. [17]. IL-6 was measured using sandwich ELISAs from R and D Systems (Quantikine PTA00 and Duoset DY686, respectively). All samples were tested in duplicate, with detection limits of 70.8 ng/mL (CRP), 31.25 µg/mL (SAA), and 62.5 pg/mL (IL-6). Blood samples for bacterial culture (5 mL) were obtained at 0 h PI (before inoculation) and at the following time points PI: 6, 12, 24, 36, and 48 h.

### Galactose elimination capacity test

After 43‒44 h post inoculation (PI), the pigs were transferred to a surgical facility for initiation of GEC test (see below). They were anaesthetised for the third time using the same premedication and anaesthetic protocol as described for the catheterisation procedure above, except that propofol dosing was adjusted to a range of 5‒10 mg/kg BW/h and supplemented with IV fentanyl (10‒30 µg/kg BW/h, Haldid^®^ 50 μg/mL; Janssen-Cilag A/S, Birkerød, Denmark). Upon anaesthesia, pigs were intubated and mechanically ventilated. An incision was made in the ventral neck followed by insertion of a catheter (Avanti catheter, 6-Fr., cat.no. 504-606X, Cordis, Fremont, CA, USA) into the carotid artery for arterial blood sampling. A urinary catheter (Foley 8 Ch, Rüsch, Kernen, Germany) was placed for collection of urine.

The GEC was performed as described by Tygstrup [18] with minor adaptations: Galactose (0.5 g/kg BW, 12 % solution; cat. no. G0750-500G, Sigma-Aldrich, St. Louis, MO, USA) was infused IV over 5 min through the right ear IV catheter. Heparin-stabilised arterial blood samples were obtained at five-min intervals in the period between 15–60 min after start of infusion. Blood samples were centrifuged within 30 min of sampling (4 °C in 10 min at 2500 rpm), and plasma was kept at −20 °C until analysis. Urine was collected for 4 h after start of galactose infusion. The total volume of urine was noted, and a sample was obtained and kept at −20 °C until analysis. Galactose plasma concentration was measured enzymatically (Pentra-400, Horiba medical) by the galactose dehydrogenase (cat. no. 10104981001, Roche, Hvidovre, Denmark) method after deproteinisation with 0.33 M perchloric acid [19]. All samples were run in duplicates. The GEC rate was calculated as described by Tygstrup [18] and Jepsen et al. [20], using the formula: GEC = (A–U)/(t_cj=0_ + 7) mmol/min, where A is the amount of galactose injected, U is the amount of galactose excreted in urine collected over 4 h, t_cj=0_ is the intercept with the time axis of a straight line fitted to the linear part of the time/arterial blood galactose concentration curve (i.e., the saturated period). The constant ‘7’ is a correction for the time-delay of galactose equilibration between extra- and intravascular spaces.

### Euthanasia

After completion of the GEC test, the pigs were euthanised by axillary exsanguination immediately following administration of a propofol overdose (3–6 mg/kg BW).

### Pathology and bacteriology

A full necropsy was performed, and tissue samples for histopathology were obtained in duplicates from hepatic lymph nodes and five pre-defined areas of the liver. Samples were fixed in 10 % formalin for 24 h, processed routinely, and cut in sections of 3–4 µm. Sections were stained with haematoxylin and eosin and blinded prior to evaluation. Presence of hyaline globules in liver sections was evaluated by haematoxylin-eosin (HE), phosphotungstic acid haematoxylin (PTAH), martius scarlet blue (MSB), and fibrinogen immunohistochemistry stains, as described previously [21].

Bacteriological examinations of *S. aureus* in tissue samples were performed as previously described [14]. Samples for bacteriology were obtained from liver, lung, spleen, bone marrow, peritoneal fluid, as well as from cases of suppurative auricular vein infection. Cotton swab samples obtained from nostrils and perineum prior to the experiment in order to check *S. aureus* colonisation status were transferred to enrichment medium consisting of 10 mL Brain Heat Infusion Broth (Oxoid CM1135) supplied with 7.5 % NaCl. After incubation at 37 °C/24 h the enrichment culture was streaked on blood agar medium (Blood agar base, Oxoid CM0055, supplied with 5 % sterile bovine blood). Haemolytic colonies were isolated and identified by Matrix-assisted laser desorption ionixation-time of flight mass spectrometry (MALDI-TOF MS) on a Vitec MS (Biomérieux) using Saramis software (Biomérieux).

## Results

### Inoculated pigs

#### Case-1 (1 × 10^7^ cfu/min/kg BW; 10 min infusion)

**Fig. 2:**
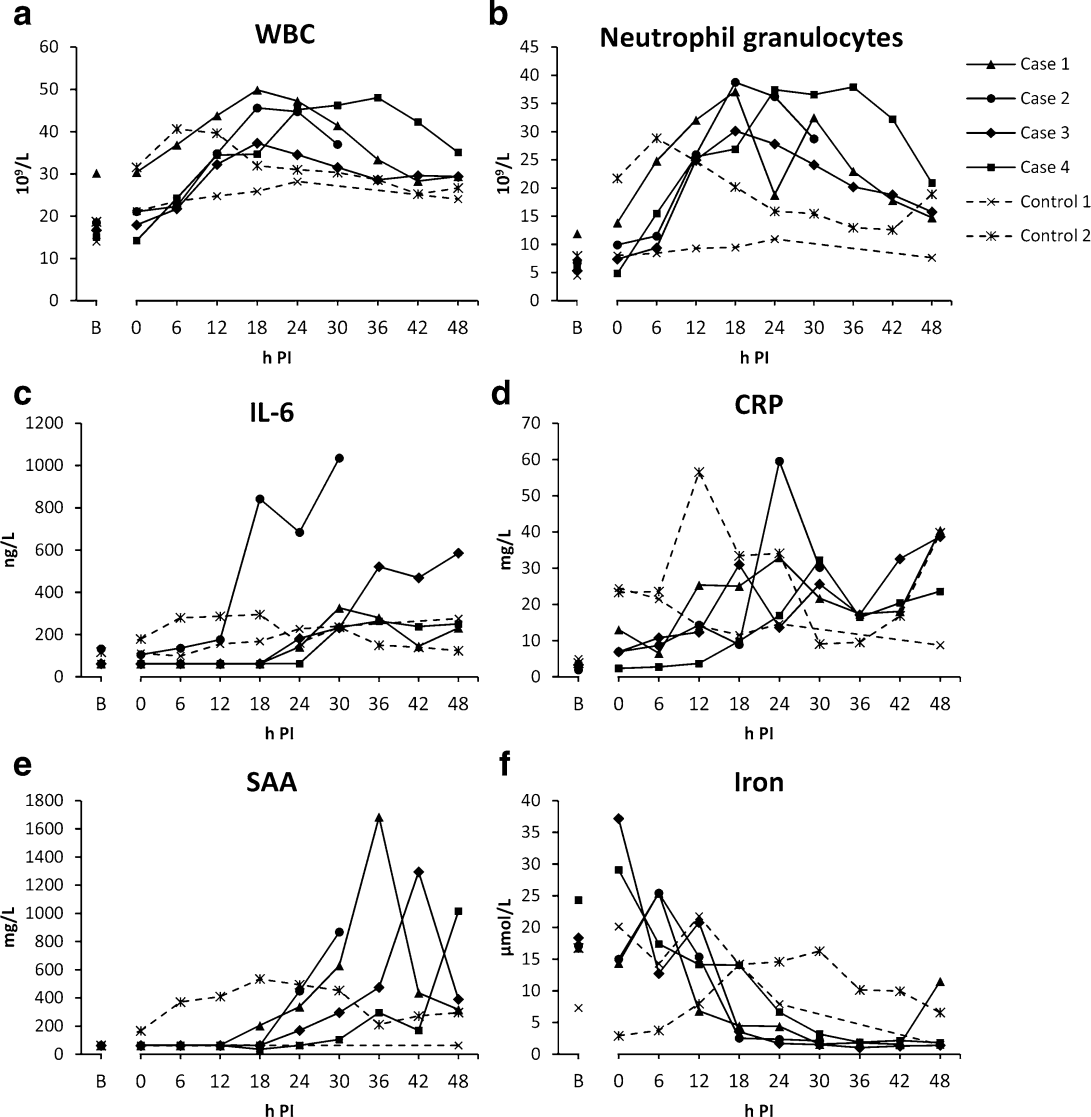
Markers of systemic inflammation. Within the group of inoculated pigs, one pig (Case-2) distinguished itself from the others by having generally an earlier onset of response, and also a more pronounced response. Blood samples were not obtained from Control-1 between 24–48 h due to a defect catheter. *B* baseline sample

**Fig. 3:**
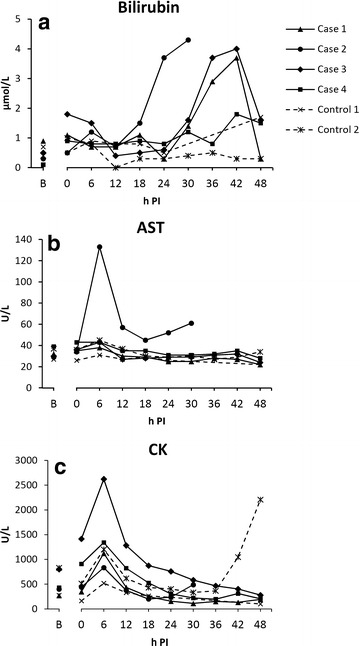
Blood parameters for assessment of hepatic function. *B* baseline sample

**Fig. 4:**
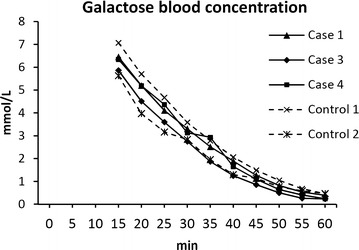
The time/arterial blood galactose concentration curve during GEC testing at 44 h PI. Slopes are identical for all pigs in the saturated, linear phase, suggesting identical galactose elimination capacities before correction for urinary output. The finding was confirmed by calculation of actual GEC rates

Only mild tachycardia during bacterial infusion (heart rate increase from 60 to 90 beats per min) was observed. The pig was agitated and restless during the first 6 h; thereafter it became recumbent and was reluctant to stand up. It became anorectic, but remained willing to drink water. Respiration was superficial and lightly elevated. Extremities became cold. This pig completed the study period without reaching any of the humane endpoints. Sepsis was indicated by increased body temperature (onset at 30 h PI), bacteraemia, and neutrophilia, together with increased IL-6, CRP, SAA, and decreased iron (Fig. 2). There were no remarkable alterations in liver blood parameters, except an increase in total bilirubin blood concentration between 24–42 h PI (Fig. 3). Pathological and bacteriological findings are summarised in Table 1. The GEC rate at 44 h PI was 1.43 mmol/min (Fig. 4).

**Table 1:** Pathological and bacteriological findings

			Gross pathology				Histology of liver		Bacteriology				
Pigs	Inoculum infusion time	Euthanised (h)	Pneumonia^a^	Nephritis^b^	Arthritis^c^	Auricular phlebitis^d^	Micro-abscesses	Hyaline globules	Blood^e^	Liver	Lung	Spleen	Bone marrow
Case-1	10 min	48	+	+	+	−	−	−	+	−	+	−	+
Case-2	3 min 20 s	30	−	+	+	−	−	+	+	−	+	−	+
Case-3	6 min 40 s	48	+	+	+	−	−	−	+	+	+	+	+
Case-4	6 min 40 s	48	+	+	−	+	−	−	+	+	+	+	+
Control-1	10 min	48	−	−	−	+	−	−	−	−	−	−	−
Control-2	3 min 20 s	48	−	−	−	+	+	−	+	−	−	−	−

^a^Acute multifocal suppurative embolic pneumonia. Estimated number of lesions ranged between 10–60

^b^Acute multifocal suppurative embolic nephritis. Number of lesions ranged from 1to 4 (bilaterally)

^c^Acute fibrinous or fibrinosuppurative arthritis. Number of affected joints observed was one or two

^d^ *S. aureus* purulent phlebitis in left ear

^e^ *S. aureus* positive cultures were obtained at following sample time points: 6, 12, 24 h for Case-1; 12 h for Case-2; 48 h for Case-3; 24, 36 h for Case-4; 12 h for Control-2

#### Case-2 (3 × 10^7^ cfu/min/kg BW; 3.33 min infusion)

Besides mild tachycardia, a brief, self-limited episode of cardiac arrhythmia occurred during bacterial infusion. This pig showed earlier and more severe clinical signs than the other pigs, i.e., it became apathetic and had muscle tremors. At 30 h PI, this pig was no longer able to stand up, and was euthanised according to the humane endpoint: “inability to get up and/or move.” Sepsis was indicated by increased body temperature (onset at 18 h PI), bacteraemia, and neutrophilia, together with increased IL-6, CRP, SAA, and decreased serum iron. Compared to the other pigs in the study, these alterations occurred earlier and/or were more pronounced (Fig. 2). Bilirubin was increased from 12 h PI and onwards (Fig. 3a). Furthermore, AST levels peaked at 6 h PI (fivefold, compared to baseline value) (Fig. 3b) and remained elevated. A similar pattern was found for CK levels (Fig. 3c), indicating that at least part of the rise in AST was due to myocyte injury rather than hepatocyte injury. Yet, when compared to the other pigs in the study, AST levels were markedly increased, while CK levels were similar to the levels of the other pigs. The extent and degree of pathological and bacteriological findings was similar to the other inoculated pigs, except that no lung lesions were noted. Histologically, aggregations of fibrin split products (hyaline globules) were found in small vessels throughout the liver (Table 1) as also seen in a subset of septic pigs in one of our previous studies [21], however fibrin exudation from small hepatic vessels was not evident. GEC was not measured in this pig due to its pre-scheduled euthanasia.

#### Case-3 and Case-4 (1.5 × 10^7^ cfu/min/kg BW; 6.66 min infusion)

Clinically, both pigs behaved in similar fashion as Case-1. Both ingested small amounts of feed during the study period, and no humane endpoints were reached. As for the other inoculated pigs, sepsis was indicated by increased body temperature (onset at 15 and 22 h, respectively), bacteraemia, and neutrophilia, together with increased IL-6, CRP, SAA, and decreased serum iron (Fig. 2). Except from a transient increase in total bilirubin between 24‒42 h in Case-3, liver parameters were unaffected (Fig. 3). Pathological and bacteriological findings resembled those of the other inoculated pigs (Table 1). Case-4 had suppurative auricular phlebitis. GEC rates at 44 h PI were 1.47 mmol/min (Case-3) and 1.79 mmol/min (Case-4) (Fig. 4).

### Control pigs

Although clinically unaffected, *S. aureus* suppurative auricular phlebitis was found in both control pigs at necropsy (Table 1). Control-2 had extensive phlegmon in relation to the phlebitis and displayed positive *S. aureus* blood culture at a single time point as well as disseminated hepatic microabscesses, while curiously, no bacteria were cultured from the liver tissue. Furthermore, altered blood inflammatory parameters were found in this pig: Neutrophilia, increased CRP, SAA, and IL-6, and decreased iron (Fig. 2). The infection most likely resided from the catheterisation procedure, as many of the parameters had started to increase prior to inoculation. None of this was seen in Control-1, however its interpretation is impeded by the lack of blood samples in the time between 24–48 h due to a defect catheter. GEC rates at 44 h PI were 1.39 mmol/min (Control-1) and 1.41 mmol/min (Control-2) (Fig. 4).

## Discussion

In this study, an optimal balance between our research objectives and animal welfare criteria were sought, but not achieved.

### Dynamic liver function measurement

Observations from our previous experimental porcine sepsis studies led to the hypothesis of liver dysfunction. Especially notable was the increase in total serum bilirubin (range of induced increase: 2–12 µmol/L [8, 9]). Bilirubin is however not a specific marker of liver dysfunction, as increases may also occur due to intravascular haemolysis. *S. aureus* possess a number of virulence factors, and of special interest in this regard is their ability to produce a number of haemolytic exotoxins in order to obtain iron from host erythrocytes. Considering the experimental setup of introducing large amounts of *S. aureus* to the blood stream, it is not unlikely that Staphylococcal haemolytic exotoxins [22] could have caused the observed increase in bilirubin levels, hereby indicating intravascular haemolysis rather than liver dysfunction. The observed fibrin leakage from hepatic vessels, on the other hand, seemed in support for the hypothesis of liver dysfunction, yet this could also be the result of a primary vascular dysfunction. In order to confirm our hypothesis of liver dysfunction, a specific, quantitative measurement of hepatic function was needed, and we therefore included the GEC test as a direct measure of the liver’s metabolic functional capacity.

As both control pigs displayed infections in and around the auricular veins, their status as true, healthy controls may be argued. Yet, since all indications of liver dysfunction were absent in both inoculated pigs and control pigs, except from Case-2, the obtained GEC rates most likely represent normal liver function. To our knowledge, only a few studies have reported GEC rates in healthy pigs; of these, our findings are within the range of those obtained in one study [23], while being a little higher (roughly estimated to one-third) than the rates obtained in two other studies [24, 25], despite the use of pigs of same breed, sex, and age. The reason for this discrepancy is currently unknown.

### Animal welfare and model robustness

The hypothesis originally formulated by Soerensen et al. [10] suggested a link between the inoculation rate and clinical response. The mechanism is not known, but perhaps rapid bacterial infusion could cause some kind of initial intoxication or extreme pulmonary hypertension that would have a negative effect on some immunological barriers, leading to enforced bacterial invasion and growth. The hypothesis was further strengthened by this study, since the pig inoculated by the highest inoculation rate (Case-2) experienced the most severe outcome and alterations in parameters similar to what had been seen in previous studies, i.e., increasing bilirubin and AST, as well as the histopathological occurrence of intravascular hyaline globules in the liver, yet without any signs of fibrin exudation. Case-2, however, became clinically affected to the extent that it had to be euthanised after 30 h, according to humane endpoints. The three remaining pigs inoculated by lower infusion rates (Case-1, Case-3, and Case-4) successfully completed the study period, but showed no obvious signs of liver dysfunction. Despite the few pigs included, this convincingly illustrates that liver dysfunction is less likely to occur without reaching any of the pre-defined humane endpoints during a 48 h study period. This is a major limitation for the use and reproducibility of the model.

This study hereby exemplifies some of the important issues and concerns of working with conscious sepsis animal models. It is inherently an ethical challenge to perform sepsis experiments in conscious animals because sepsis is a potentially fatal condition, and because the later stages of disease are of main interest for translational research [26].

In this study, we used directly determinable humane endpoints based on the knowledge gained from previous experimentation, which is crucial, especially when working with fast-evolving illness. Theoretically, researchers could be permitted to change the humane endpoints in order to allow further disease progression, if the sought knowledge is important enough to compensate for animal suffering, and as long as it cannot be achieved by other means. Another solution is to use long-term anaesthesia. While being the most humane solution, long-term anaesthesia however has the disadvantage of affecting the inflammatory processes and disease progression [27]. On the other hand, long-term sedation or anaesthesia is frequently used in the management of patients with severe sepsis in ICUs; thus, mimicking such a clinically relevant setting may be the most optimal choice for further development of this porcine sepsis model.

## Conclusions

Re-construction of signs of liver dysfunction could not be achieved within the limits of our pre-defined humane endpoints. In light of this result, a usable balance between scientific purpose and animal welfare appears unfeasible, and we find it hard to justify any further use of this conscious porcine sepsis model. We suggest that this model should be re-established as a long-term anaesthesia model in order to make a model of translational relevance for human sepsis.

## Abbreviations

ALT: alanine aminotransferase
AP: alkaline phosphatase
AST: aspartate aminotransferase
CK: creatine kinase
CRP: C-reactive protein
GEC: galactose elimination capacity
HE: haematoxylin-eosin
MSB: martius scarlet blue
PI: post inoculation
PTAH: phosphotungstic acid haematoxylin
SAA: serum amyloid A
WBC: white blood cell count
